# Alpha-Lipoic Acid Is an Effective Nutritive Antioxidant for Healthy Adult Dogs

**DOI:** 10.3390/ani11020274

**Published:** 2021-01-22

**Authors:** Reshma M. Anthony, Jennifer M. MacLeay, Dennis E. Jewell, John J. Brejda, Kathy L. Gross

**Affiliations:** 1 Hill’s Pet Nutrition, Inc., 1035 NE 43rd Street, Topeka, KS 66617, USA; jen_macleay@hillspet.com (J.M.M.); kathy_gross@hillspet.com (K.L.G.); 2 Department of Grain Science and Industry, Kansas State University, Manhattan, KS 66506, USA; djewell@ksu.edu; 3 Alpha Statistical Consulting, 1220 South 25th, Lincoln, NE 68502, USA; alphastatistical01@gmail.com

**Keywords:** alpha-lipoic, acid, nutritive antioxidant, glutathione, healthy adult dogs

## Abstract

**Simple Summary:**

A 6 month prospective, controlled clinical trial was conducted to determine the nutritive antioxidant activity of alpha-lipoic acid as a maintenance food for healthy adult (non-gestational, non-lactating) dogs. The results of the study showed that increasing concentrations of alpha-lipoic acid induced a significant increase in the concentrations of intracellular glutathione, an important biomarker of the antioxidant system.

**Abstract:**

This study was designed to determine the effect of alpha-lipoic acid on the glutathione status in healthy adult dogs. Following a 15 month baseline period during which dogs were fed a food containing no alpha-lipoic acid, dogs were randomly allocated into four groups. Groups were then fed a nutritionally complete and balanced food with either 0, 75, 150 or 300 ppm of alpha-lipoic acid added for 6 months. Evaluations included physical examination, body weight, food intake, hematology, serum biochemistry profile and measurements of glutathione in plasma and erythrocyte lysates. Throughout, blood parameters remained within reference ranges, dogs were healthy and body weight did not change significantly. A significant increase of 0.05 ng/mL of total glutathione in red blood cell (RBC) lysate for each 1 mg/kg bodyweight/day increase in a-LA intake was observed. In addition, a significant increase was observed for GSH, GSSG and total glutathione in RBC lysate at Month 6. We conclude that alpha-lipoic acid, as part of a complete and balanced food, was associated with increasing glutathione activity in healthy adult dogs.

## 1. Introduction

Alpha-lipoic acid (often called α-lipoic acid and abbreviated here as a-LA), is a naturally occurring organosulfur compound synthesized by plants and animals, including humans [1,2]. It contains two thiol (sulfur) groups, which may be oxidized or reduced; dihydrolipoic acid (DHLA) is the reduced form of lipoic acid [3]. a-LA has many roles in body processes, including a pivotal role in energy metabolism as a cofactor for α-keto acid dehydrogenases in mitochondria [4]. Endogenous lipoic acid is assumed to be sufficient for metabolism, but supplementing a-LA in food may confer additional benefits, and it has been suggested that it may be conditionally essential in aged animals [5].

a-LA and its reduced form have been shown to possess physiological activity as an antioxidant, and properties contributing to cardiovascular and cognitive health, anti-aging, detoxification, anti-inflammation, anti-cancer and neuroprotection [6,7]. a-LA has been reported to have beneficial effects in humans in disease states such as diabetes, dementia and multiple sclerosis. Several clinical trials in individuals with type 2 diabetes show that high doses of a-LA improve glucose utilization, improve insulin sensitivity and lower fasting blood glucose and insulin concentrations [8,9,10,11,12,13]. Clinical trials show that intravenous or oral administration of a-LA improves symptoms of diabetic peripheral neuropathy [14,15,16]. a-LA supplementation improves glucose metabolism and insulin resistance in rats [17,18]. a-LA is also found to reduce blood pressure in rats with hypertension [19]. a-LA has been proven to reduce oxidative stress in rats and canines [20,21] and function as an anti-inflammatory agent in mice [22,23]. Studies indicate that a-LA improves neurocognitive function in rodent and canine models [24,25,26]. a-LA is unique as it is soluble in water or lipids and can function as an antioxidant inside and outside cells [7]. It can function as an antioxidant in virtually all tissues and therefore may be referred to as a universal antioxidant or the antioxidant of antioxidants [6,7,27,28]. The molecule functions as an antioxidant through several different mechanisms including scavenging free radicals, chelating metals and contributing to the repair of damaged proteins and lipids [6,29,30]. a-LA scavenges free radicals and is involved in the regeneration of other antioxidants such as glutathione, vitamin E, vitamin C, and coenzyme Q10 [2,29,31]. It can regenerate ascorbic acid from dehydroascorbic acid and can indirectly regenerate vitamin E from its oxidized state [28]. a-LA chelates metals and has been shown to effectively chelate toxic metals directly, as well as indirectly by its ability to increase glutathione, which in turn functions as a metal chelator [7,27,28,29,32,33]. Metals such as manganese, zinc, cadmium, lead, cobalt, nickel, iron and copper are known to form complexes with a-LA and DHLA [7,28,32,33]. Such complexation of metals by a-LA and DHLA may provide antioxidant activity. a-LA has been shown to chelate and reduce iron and consequently protect against peroxidation of lipids [34]. Similarly, complexation of Cu^2+^ by a-LA explains the protection in Cu^2+^-induced lipid peroxidation [33]. a-LA prevented Cd^2+^-induced lipid peroxidation in brain, heart and testes of rats [35]. Lastly, a-LA functions as an antioxidant by repairing damaged molecules of physiological structures such as DNA, lipids and proteins [7,27].

Lipoic acid is readily available as a dietary supplement in both human and veterinary markets. Lipoic acid can be absorbed from foods or dietary supplements. However, it has been shown that the bioavailability of lipoic acid in humans and dogs is decreased when administered as a supplement concomitant with food or from a natural food source [36,37]. As a nutritive, a-LA is provided at a dose far less than the reported oral LD_50_ of 400 to 500 mg/kg bwt for dogs [38]. The maximum tolerated dose of a-LA in dogs was found to be 126 mg/kg bwt [39], and a case report of two dogs showed that clinical signs of acute toxicity and death occurred at estimated single oral doses of 190 mg/kg and 210 mg/kg body weight, respectively [31]. Symptoms of lipoic acid toxicity in dogs include vomiting, ataxia, tremors, seizures, hypersalivation, lethargy and weakness [31]. Lower doses have been found to be beneficial. A single dose of lipoic acid was administered to dogs at 10 to 48 mg/kg bwt orally or by subcutaneous/intraperitoneal routes [40,41] and appeared to protect dogs from arsenite toxicity [41]. In Zicker et al. [21], the safety and efficacy of a-LA were evaluated in 30 adult beagles, which were evenly randomized into five groups, each of which was fed one of five different foods with varying concentrations of a-LA (0, 150, 1500, 3000 and 4500 ppm of food). This corresponded to an exposure of a-LA in the range of 0 to 85 mg/kg bwt. The investigators documented a positive effect on the GSH:GSSG (reduced glutathione: oxidized glutathione) ratio, an important biomarker of oxidative stress at the lowest level of a-LA (150 ppm food or approximately 2.7 mg/kg bwt) and showed a wide margin of safety that had not been previously reported. An additional 6 months of data was published by Pateau-Robinson et al. in 2013 [42] and included a total of 12 months of feeding compared to the original 6 months published by Zicker et al. [21]. This study demonstrated that the inclusion of a-LA up to 3000 ppm in dog food did not present any health risks. Antioxidant activity was not further evaluated in months 7–12. The current study was conducted to determine the antioxidant activity of a-LA involving a larger population of dogs in order to support its use in foods, on a daily basis, for healthy adult (non-gestational, non-lactating) dogs. The concentrations of a-LA used were 0, 75, 150 and 300 ppm, which corresponded to an exposure between 0 and 5 mg/kg body weight. As an antioxidant effect was seen in Zicker et al. [21] at 2.7 mg/kg body weight, 150 ppm was used for one group to see if the same effect could be repeated in a larger and more diverse group of animals in the current study. A concentration of 75 ppm (1.2 mg/kg bwt) was used to determine if the antioxidant effect would be seen at a lower concentration than 150 ppm, and 300 ppm was used to see if the effect was enhanced at twice the concentration. The current study consisted of a long baseline period of 15 months compared to the 2 week period in Zicker et al. [21] to minimize the effects of confounding factors. The antioxidant activity of a-LA was determined by measuring the intracellular levels of GSH (reduced glutathione) and GSSG (oxidized glutathione). GSH is a major intracellular, water-soluble antioxidant, and GSH homeostasis is critical in protecting cells from oxidative stress [43,44]. GSH, the ratio of GSH with oxidized glutathione (GSSG) and the levels of total glutathione are used as markers of oxidative stress [45,46,47]. The ability of a-LA to improve intracellular levels of GSH has been reported in several studies [48,49]. In the current study, the change of reduced glutathione (GSH), oxidized glutathione (GSSG), total glutathione (GSH + GSSG) and the ratio (GSH/GSSG) over time was analyzed as a function of average lipoic acid (LA) intake, in mg/kg body weight/day.

## 2. Experimental Section

### 2.1. Study Design

All methods were approved by appropriate Animal Care and Use Committees and adhered to the Sponsor’s Animal Welfare Policy. This study consisted of two phases: Phase 1 lasting 15 months and Phase 2 lasting 6 months. During Phase 1, 92 dogs, verified as healthy based on complete blood count (CBC), serum chemistry, urinalysis and physical examination, were fed a complete and nutritionally balanced maintenance food without added lipoic acid. This phase was done to minimize residual effects of varying intake of dietary antioxidants such as Vitamin E within the population. The food fed in Phase 1 was similar to the food used for Phase 2, except for the inclusion of varying levels of a-LA.

For phase 2, 80 dogs were randomly selected from the 92 dogs participating in Phase 1 and randomized to 1 of 4 feeding groups of 20 dogs each. Foods were formulated to contain 0 ppm, 75 ppm, 150 ppm and 300 ppm a-lipoic acid and were fed for 6 months. The animals were blocked for age and Vitamin E status at the start of Phase 2. The animals were not blocked on body weight or on gender. Bodyweight was not a reflection of the body condition score (BCS) but a reflection of the physical size of the animal. All dogs were fed their respective foods daily as the sole source of nutrition. The recipe details and nutrient composition of the study foods are shown in Appendix A (Table A1 and Table A2), respectively. The control and test foods were analyzed for lipoic acid prior to the start of the study and also during the study to ensure that target supplementation levels were achieved (Appendix A Figure A3). Intake of lipoic acid (mg/kg body weight) was calculated by using the mean food intake (in kg) times the lipoic acid content (in ppm; as fed) divided by the animal’s body weight (kg). The mean lipoic acid exposure for the different treatment groups was calculated to be 0, 1.20, 2.7 and 4.94 mg/kg bodyweight/day.

Evaluations included physical examinations to assess overall health of animals, hematology and serum biochemistry profiles (months 0 and 6), bodyweight (every other week), food intake (daily), and measurements of reduced glutathione, oxidized glutathione levels and total glutathione levels as well as the ratio of reduced glutathione to oxidized glutathione in both plasma and erythrocyte lysates of all animals (months 0, 2, 4 and 6). Values for hematology and serum biochemistry were compared to normal canine values to help determine overall health.

### 2.2. Test Animals and Feeding

Dogs between ages 5–14 years of age at study start were used (Table 1). There were no gender specifications, both male and female, spayed and neutered were included. Various purebred and mixed breed dogs with an initial minimum body weight of 5 kg with Body Condition Score (BCS) of 2–4/5 or Body Fat Index of ≥20 were included in the study. Dogs were excluded from the study if they were obese (had BCS > 5), had been diagnosed with a chronic disease condition, including, but not limited to kidney disease, hypothyroidism, or diabetes. Dogs enrolled in the study could be removed from the study if that dog (1) experienced excessive weight loss (dogs with weight loss of >15% of initial body weight were examined for health and removed from the study); (2) stopped eating for 2 days or ate less than 50% of food ration for 3 days; or (3) was diagnosed with any secondary systemic disease such as diabetes, hypothyroidism or chronic kidney disease.

All study animals were allowed normal socialization and enrichment activities, both indoors and outdoors, and the design of the study did not interfere with the animals’ normal daily routine. Dogs had water offered ad libitum, and daily food intake was recorded. The food fed in Phase 1 contained 0 ppm a-LA added and met or exceeded all nutrient recommendations for maintenance of adult dogs as proposed by the Association of American Feed Control Officials [50]. The foods fed in Phase 2 were identical with the exception of the addition of varying amounts of a-LA. At the beginning of each study phase, each dog was weighed every 2 weeks for 10 weeks and then approximately every 4 weeks for the duration of that phase. The amount of food fed was determined using metabolic body weight [21,42]. ((Metabolic body weight = (bodyweight)^0.75^. Resting Energy Requirements (RER) is calculated by multiplying this metabolic weight by 70. Metabolic weight is only indicative, and the caloric requirement depends on the activity level of the dogs, BCS and other factors.) The amount of food was adjusted to maintain each dog’s ideal body weight as necessary. Each dog received a complete physical exam, blood work for CBC and serum chemistry, and urinalysis to rule out occult systemic disease at the beginning and at the end of Phase 1. All dogs were healthy on physical exam. Blood work for CBC and serum chemistry analysis was done again at the end of Phase 2. In addition to this, blood work for glutathione measurements was done at Months 0, 2, 4 and 6 of Phase 2.

### 2.3. Concentrations of Alpha-Lipoic Acid in Food

Concentrations of alpha-lipoic acid (ppm) in food were determined periodically over 72 weeks (Week 0, 8, 16, 24, 32, 40, 48, 56, 64 and 72) by high-performance liquid chromatography [37] and were within the range of expected assay sensitivity and production parameters (Appendix A Figure A3).

### 2.4. Glutathione Analytical Methods

The biomarker glutathione was used to demonstrate the nutritive antioxidant property of a-LA. Evaluation of glutathione included reduced glutathione (GSH) and oxidized glutathione (GSSG) in plasma and red blood cell lysate. High-performance liquid chromatography coupled with tandem Mass Spectrometric detection (HPLC–MS/MS) was applied to quantitate the amount of GSH and GSSG in plasma and red blood cell lysate.

The plasma samples were treated with 10% meta-phosphoric acid (MPA) to precipitate the proteins. After shaking and centrifugation, the supernatant was appropriately diluted with 10% MPA and spiked with an internal standard, tiopronin. The matrix standards were prepared in the same manner, then spiked with calibration standards, GSH-^13^C_2_,^15^N_1_ and GSSG-^13^C_4_,^15^N_2_. Labeled forms of GSH and GSSG were used as calibration standards for accurate measurement as GSH and GSSG already existed in blank canine plasma. For the calibration curve, standards were spiked into blank canine plasma, and the sample was quantified against this curve.

The red blood cell (RBC) lysate samples were treated with 10% meta-phosphoric acid (MPA) to precipitate the proteins. The obtained supernatant was appropriately diluted with a solution containing 0.1% formic acid, 3% MPA, 1 mM disodium ethylenediaminetetraacetate dihydrate (EDTA-Na_2_). This diluted supernatant was spiked with an internal standard (IS), GSH-^13^C_2_,^15^N_1_ and GSSG-^13^C_4_,^15^N_2_. For the calibration, the surrogate matrix (0.2% formic acid, 3% MPA, 1 mM EDTA-Na_2_) containing IS was used after being spiked with calibration standards, GSH and GSSG, and analyte concentrations in samples were quantified against these curves. A surrogate matrix was used for the calibration curve in this study since GSH and GSSG are endogenous in blank canine RBC lysate.

The LC–MS/MS method was developed by using a C-18 based column for HPLC separation on a Shimadzu LC-20AT system (Shimadzu Scientific Instruments, Columbia, MD, USA), and the compound was separated using 0.1% formic acid in water (A) and 0.1% formic acid in methanol (B) in gradient mode. The compounds were detected in the positive-ion-mode multiple reaction monitoring (MRM) MS/MS method for compound quantification with a SCIEX 4000 QTRAP System, a triple quadrupole mass spectrometer. The daughter ions of compounds were determined by fragmentation experiments, and the most abundant daughter ions were selected for compound quantifications. The developed method was applied to quantify GSH and GSSG. GSH:GSSG was determined by using the amount of GSH divided by the amount of GSSG. Total glutathione was determined by adding the amount of GSSG and the amount of GSH together.

### 2.5. Statistical Analysis and Methods

All statistical analyses were performed using SAS^®^, version 9.4 (https://www.sas.com/en_us/home.html). Differences in animal age between the four foods were analyzed using a one-way analysis of variance (ANOVA) with food as the only fixed-effect using PROC GLM. Differences in animal weight and intake between the four foods were analyzed using a heterogeneous variance ANOVA model with food as the only fixed-effect using PROC MIXED. This was done because variances for animal weight and intake were unequal for the four foods. Distribution of gender was analyzed using Fisher’s exact test.

Serum chemistry and CBC profiles were summarized (n, mean, standard deviation, minimum, median and maximum) for each food at Month 0 and Month 6 in the test phase using PROC MEANS. In addition, change from baseline (CFB) values were calculated by subtracting Month 6 values from Month 0 values. A one-sample *t*-test was used to determine if the CFB values were significantly different from 0 at the alpha = 0.10 probability level using PROC TTEST. In addition, the same summary statistics described above and a 90% confidence interval were calculated for the CFB values. All analyses were performed on the intent-to-treat population.

The distribution of total glutathione concentrations as well as GSH and GSSG fractions were examined and found to be strongly right-skewed, with variances increasing with the means. Therefore, a natural-log (ln) transformation was used to stabilize the variances and provide a more normal distribution to the data. Because all concentration values were positive and non-zero, no integer was added to the values prior to transformation.

Intake was measured at daily intervals, but glutathione was measured at bimonthly intervals at 0-, 2-, 4- and 6-month time points. The average lipoic acid intake was calculated for the bimonthly interval leading up to the glutathione sample point. This was done to provide an estimate of average lipoic acid intake to match with each glutathione sampling point [21]. Because there were endogenous levels of glutathione in the dogs at month 0, but no lipoic acid intake prior to that time point, the Month 0 time point was defined to be a baseline value, and a CFB calculation in total glutathione and GSH and GSSG fractions was made at Months 2, 4 and 6, using ln-transformed values. The CFB in ln-transformed glutathione values were regressed against average lipoic acid intake over all sampling points to examine the relationship between lipoic acid intake and glutathione concentrations in the plasma and RBC lysate(https://www.sas.com/en_us/home.html). A random animal effect was included in the linear model to account for animal-to-animal variation, and a fixed month effect was included in the model to account for month-to-month variation. All analyses were performed using SAS^®^ PROC MIXED(https://www.sas.com/en_us/home.html).

Bodyweight data were analyzed using a linear mixed model with Food, Day and Food × Day as fixed effects. The first-order ante-dependence [ANTE(1)] covariance structure was used to model the correlation between the repeated measures. This covariance structure was selected based on the AICC fit statistics. The Kenward–Roger adjustment (DDFM = KR) was used to calculate the denominator degrees-of-freedom for the fixed effects. Animals’ weights for each food during the test period were compared against weights at the start of the test period using the SLICEDIFF option with the Dunnett adjustment to control the Type I error rate. The analysis was performed using SAS^®^ PROC GLIMMIX(https://www.sas.com/en_us/home.html).

Average intake data for each Food was analyzed using a linear mixed-model with Food, Period and Food x Period as fixed effects. The first-order autoregressive heterogeneous [ARH(1)] covariance structure was used to model the correlation between the repeated measures. This covariance structure was selected using the AICC fit statistic. The Kenward–Roger adjustment (DDFM = KR) was used to calculate the denominator degrees-of-freedom for the fixed-effects. Average intake in Periods 2 through 6 was compared to Period 1 using a set of estimate statements. The adjust-simulate option was used to control the Type I error rate. The analysis was performed using SAS^®^ PROC GLIMMIX(https://www.sas.com/en_us/home.html).

## 3. Results

Patient signalment and food group information are summarized in Table 1. Twenty animals were assigned to each group. One animal in the Food 3 (300 ppm a-LA) group did not complete the study and was euthanized due to anaplastic sarcoma, which was not study-related (Table 2). All data for this animal until its death were included in the analysis. Overall, there were no significant differences between groups with respect to gender, age or baseline vitamin E status. The mean body weight for dogs consuming Food 3 was significantly lower than the other three groups at study start. However, there was no significant change in bodyweight of animals within groups during the study (Table 3).

The mean food intake of the animals of each group in month/period 2, 3, 4, 5 and 6 was compared to the intake in month/period 1 of the respective groups. There was no significant change in food intake of animals within each group during the study (Table 4).

CBC/Serum chemistry: Differences in blood measurements were measured between the start and the end of the feeding phase of the study for each Food group. All data were within the lab’s reference ranges, and all animals were free of overt clinical disease. Several significant but not clinically significant differences were noted and are detailed in Appendix A (Table A3 and Table A4). Differences noted were neither large nor outside reference ranges and were deemed to be not clinically important.

Relationship between Glutathione and a-LA intake: A significant change was observed with GSH and total glutathione (GSH + GSSG) in RBC lysate when the analysis was pooled over all time points. There was a significant increase of 0.049 ng/mL of GSH and 0.047 ng/mL of total glutathione in RBC lysate for each 1 mg/kg body weight/day increase in a-LA intake (Table 5). Significant changes were not observed in total glutathione levels in plasma. A significant change was observed for GSH, GSSG and total glutathione in RBC lysate at Month 6, when the time points were analyzed separately (Table 6).

## 4. Discussion

This study verified the utility of the nutrient additive a-LA as an antioxidant in healthy adult dogs. There was a significant increase of 0.049 ng/mL of GSH and 0.047 ng/mL of total glutathione in RBC lysate for each 1 mg/kg body weight/day increase in a-LA intake. The daily consumption of dry dog food containing up to 300 ppm a-LA for 6 months did not have any adverse effects on physical appearance, body weight, food intake, serum biochemistry or CBC of healthy adult dogs. The average lipoic acid intake for the different treatments groups was calculated to be 0, 1.20, 2.7 and 4.94 mg/kg body weight/day. Intakes, despite normal variation in population body weights, were much lower than the maximum tolerated dose of a-LA in dogs of 126 mg/kg bwt [39] or the 400–500 mg/kg body weight LD50 [38].

**Figure A1:**
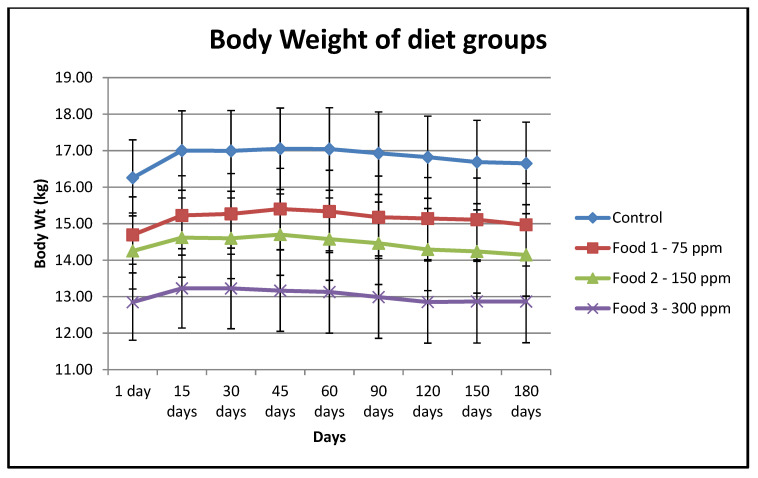
Bodyweights of animals in all food groups during the test phase of the study. Control, Food 1, Food 2 and Food 3 have 0 ppm, 75 ppm, 150 ppm and 300 ppm of a-LA respectively.

**Figure A2:**
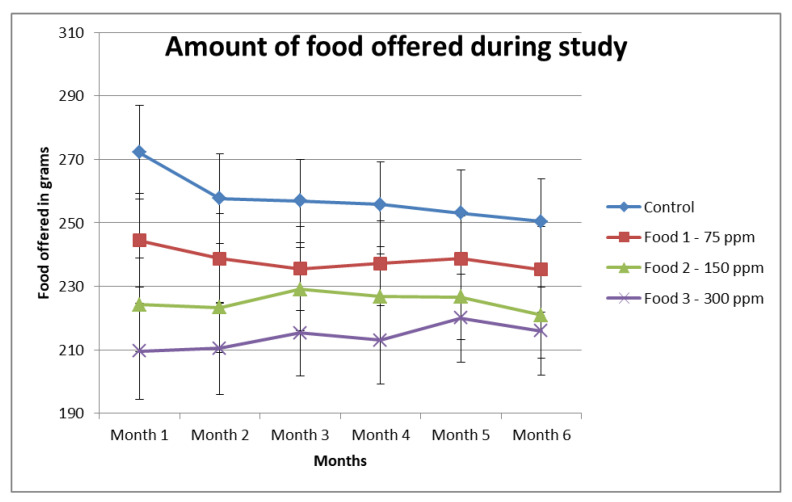
Food offered to animals in all food groups during the test phase of the study. Control, Food 1, Food 2 and Food 3 have 0 ppm, 75 ppm, 150 ppm and 300 ppm of a-LA respectively. The amount of food offered was adjusted in response to weight gain at the end of weeks 2, 4, 6 and/or 8 and thereafter, on an individual basis, and accounts for decreased food offered to maintain body weight.

**Figure A3:**
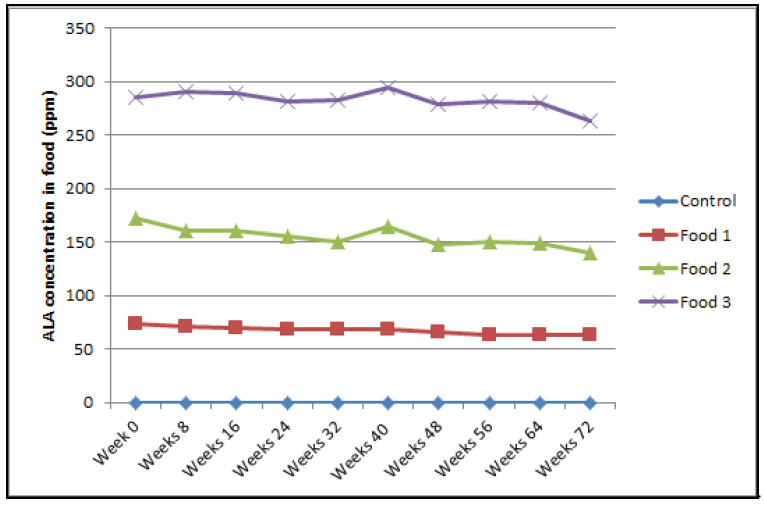
Mean a-LA concentration in ppm in the different Foods analyzed by High-Pressure Liquid Chromatography Control, Food 1, Food 2 and Food 3 have 0 ppm, 75 ppm, 150 ppm and 300 ppm of a-LA respectively.

An interesting finding was that at the beginning phase 2, animals in all four groups started gaining weight, with the control group gaining the most and the highest inclusion group gaining the least (Appendix A Figure A1), suggesting improved feed efficiency. To avoid excessive weight gain, the quantity of the food was adjusted to maintain body weight (Appendix A Figure A2). Ultimately, the dogs did not gain more weight than those in the other inclusion groups. This could potentially be explained by the ability of a-LA to promote body weight and fat mass loss by increasing energy expenditure [51,52] and lipolysis [53], improving insulin sensitivity [54] and inhibiting lipogenesis [53].

Of the adverse events observed in this study (Table 2), none of them was attributed to the food. The GI complications observed in some animals were not secondary to a-LA supplementation in food because they resolved while the study diets continued. Sporadic health events were not study-related, including the one death due to anaplastic sarcoma. This animal’s data, until its death, were used for statistical analysis. Daily observation of the dogs did not reveal any clinical signs of toxicity in any of the dogs during the course of the study. Serum biochemistry and hematology were monitored regularly throughout the study and were interpreted not to be clinically significant. The values of all blood parameters stayed within normal laboratory reference ranges.

Lipoic acid has been reported to have several biologic effects in humans and animals. One of its many roles includes activity as a direct antioxidant by regeneration of major physiological antioxidants, such as Vitamin E, ascorbic acid, and GSH [2,27,31]. Glutathione is a major intracellular, water-soluble antioxidant, and GSH homeostasis plays a vital role in defending tissues against oxidative stress [43,44]. Therefore, GSH availability is integral to the detoxification of free radicals produced by the mitochondrion. Recent studies have shown the mechanism by which a-LA increases cellular GSH [43,44,48,49]. Addition of a-LA is proposed to reduce cystine to cysteine, which is more easily transported into the cell, resulting in accelerated GSH synthesis [43,55]. In Zicker et al. [21], the addition of lipoic acid at 150 ppm (approximately 2.7 mg/kg body weight) to dogs’ food showed maximal improvement in the GSH:GSSG ratio (ratio of reduced:oxidized glutathione) in lymphocytes with a wide margin of safety in dogs. Another study showed that a-LA supplementation in rats significantly increased total GSH levels in the liver (*p* < 0.001) and blood (*p* < 0.05) [56]. Our study showed a significant increase in GSH, GSSG and total glutathione in RBC lysate with increasing concentrations of a-LA intake, but such significant increases were not observed in plasma, indicating that intracellular biomarkers may be better indicators of a-LA activity than plasma biomarkers. In fact, erythrocytes have a remarkably high activity of GSH synthesizing enzymes [57,58], and it has been shown that GSH disappears rapidly in plasma, whereas GSSG disappears relatively slowly [59]. Although most of the glutathione in plasma is in the form of GSH, it is the major source of thiol in plasma and is constantly translocated across cell membranes and used in reactions, which leads to formation of GSSG and other products [59,60,61,62]. Moreover, glutathione reductase, the enzyme involved in reduction of GSSG to GSH, occurs only intracellularly, and there is no extracellular mechanism for GSSG reduction. Whether GSH in plasma reacts to form other types of glutathione derivatives remains to be explored.

Studies in humans and other species have shown that sexual dimorphism could have an impact on GSH metabolism [63]. The report states that these differences could be due to genetic factors, levels of oxidative stress and sex hormones. The animals in the current study were blocked on age and Vitamin E status and not on gender. With sexual differences in glutathione metabolism observed in several studies in the report [63], not blocking the animals on the basis of gender could be a limitation of the study. Most of the dogs in the study were neutered and spayed; therefore, sex hormones are unlikely to be a confounding factor although genetic factors could still play a role. The observed antioxidant effect of supplemental a-LA on intracellular glutathione paves a path to explore its effect on other biomarkers of the glutathione system such as glutathione related enzymes namelyglutathione peroxidase and glutathione reductase. Furthermore, the investigation can be extended to study the antioxidant effect of a-LA on a broad profile of endogenous antioxidant biomarkers such as superoxide dismutases, peroxiredoxins, heme oxygenases, catalases and products of lipid peroxidation.

## 5. Conclusions

This study demonstrated the effect of dietary a-LA on glutathione status in dogs. The inclusion of a-LA in dog foods in this study posed no health risks and improved levels of the endogenous antioxidant glutathione in a dose-related fashion, showing the antioxidant effect of a-LA. The study thereby supports the use of a-LA as a safe and effective nutritive antioxidant in foods for healthy, non-gestational, not lactating adult dogs.

## Figures and Tables

**Table 1:** Patient characteristics at the study start for each group. Data are represented as absolute counts or as average ± standard deviation. (Control, Food 1, Food 2 and Food 3 have 0 ppm, 75 ppm, 150 ppm and 300 ppm of a-LA respectively.)

Patient Characteristics	Overall	By Group			
		Control	Food 1	Food 2	Food 3
Number of Animals	80	20	20	20	20
Male	3	1	1	1	0
Female	1	0	1	0	0
Neutered	41	9	9	9	14
Spayed	35	10	9	10	6
Mean age (yr)	10.2 ± 1.8	9.8 ± 2.1	10.0 ± 1.6	10.6 ± 1.8	10.5 ± 1.7
Mean weight (kg)	14.6 ± 1.0	16.3 ± 1.0	14.7 ± 1.0	14.3 ± 1.0	12.9 ± 1.0
Mean serum Vitamin E (μg/mL)	33.8 ± 8.2	35.0 ± 8.8	33.9 ± 9.0	34.0 ± 8.1	32.2 ± 7.1

**Table 2:** Adverse events are summarized below. (Control, Food 1, Food 2 and Food 3 have 0 ppm, 75 ppm, 150 ppm and 300 ppm of a-LA respectively).

Test	Food Group (If Applicable)	Description of Adverse Event	No of Animals
Phase 1	N/A	Loose stool observed and resolved	1
Phase 1	N/A	Perineal hernia, not study-related	1
Phase 1	N/A	Hemangiosarcoma, not study-related	1
Phase 2	Food 1	Vomiting/anorexia—hepatopathy, resolved, completed study	1
Phase 2	Food 3	Coughing × 2 days—age-related cardiac disease, not food-related. Started on diuretics, completed study	1
Phase 2	Food 3	Anima died, due to anaplastic sarcoma, not study-related	1

**Table 3:** Mean Body weights (in kgs) of each group, at the beginning and at end of the test phase. (Control, Food 1, Food 2 and Food 3 have 0 ppm, 75 ppm, 150 ppm and 300 ppm of a-LA respectively.)

Food	n	Mean Body wt (In kgs) of Dogs in Each Group on Day 1	SE	Mean Body wt (In kgs) of Dogs in Each Group on Day 180	SE	Adjusted *p*-Value of Mean Body wt Day 1 vs. 180
Control	20	16.26	1.04	16.65	1.13	0.53
Food 1	20	14.69	1.04	14.97	1.13	0.68
Food 2	20	14.25	1.04	14.14	1.13	0.96
Food 3	19	12.85	1.04	12.87	1.13	1.00

**Table 4:** Mean food intake by 30 day periods for all groups, difference in mean and standard error (SE) food intake between groups during every period/month of the test phase. One animal in Food 3 group was euthanized in Period 4 and hence for Food 3, n = 19 for Periods 5 and 6. (Control, Food 1, Food 2 and Food 3 have 0 ppm, 75 ppm, 150 ppm and 300 ppm of a-LA respectively.)

Food	Period/ 30 Days	n	Mean (g)	SE	Difference vs. Period 1	Adjusted *p*-Value vs. Period 1
Control	1	20	272.2	14.8		
Control	2	20	257.7	14.1	−14.5	0.22
Control	3	20	256.9	13.1	−15.4	0.28
Control	4	20	255.8	13.4	−16.4	0.27
Control	5	20	253.1	13.5	−19.2	0.27
Control	6	20	250.4	13.5	−21.8	0.27
Food 1	1	20	244.4	14.8		
Food 1	2	20	238.7	14.1	−5.7	0.49
Food 1	3	20	235.6	13.1	−8.8	0.46
Food 1	4	20	237.2	13.4	−7.2	0.55
Food 1	5	20	238.7	13.5	−5.7	0.71
Food 1	6	20	235.3	13.5	−9.1	0.57
Food 2	1	20	224.2	14.8		
Food 2	2	20	223.3	14.1	−0.9	0.99
Food 2	3	20	229.1	13.1	4.9	0.68
Food 2	4	20	226.8	13.4	2.6	0.90
Food 2	5	20	226.7	13.5	2.5	0.94
Food 2	6	20	220.9	13.5	−3.3	0.91
Food 3	1	20	215.1	14.9		
Food 3	2	20	213.5	14.1	−1.6	0.93
Food 3	3	20	219.9	13.2	4.8	0.71
Food 3	4	20	214.0	13.4	−1.1	0.99
Food 3	5	19	220.9	13.5	5.8	0.75
Food 3	6	19	216.8	13.5	1.8	0.99

**Table 5:** Relationship between a-LA intake (in mg/kg body weight/day) and glutathione levels (in ng/mL) pooled over months 2, 4 and 6 in plasma and RBC Lysates.

		Slope		95% Confidence Interval		
Matrix	Analyte	Estimate	SE	*p*-Value	Lower	Upper
Plasma	GSH	−0.011	0.037	0.774	−0.084	0.063
Plasma	GSSG	−0.014	0.041	0.726	−0.095	0.066
Plasma	GSH/GSSG Ratio	0.005	0.031	0.880	−0.057	0.067
Plasma	Total glutathione	−0.014	0.036	0.709	−0.086	0.059
RBC Lysate	GSH	0.049	0.021	0.024	0.01	0.092
RBC Lysate	GSSG	0.043	0.034	0.203	−0.024	0.110
RBC Lysate	GSH/GSSG Ratio	0.006	0.029	0.846	−0.052	0.063
RBC Lysate	Total glutathione	0.047	0.021	0.029	0.005	0.089

**Table 6:** a-LA intake (in mg/kg body weight/day) and glutathione levels (in ng/mL) in plasma and RBC Lysates at Month 6 vs. baseline.

z		Slope		95% Confidence Interval		
Matrix	Analyte	Estimate	SE	*p* Value	Lower	Upper
Plasma	GSH	0.021	0.054	0.694	−0.086	0.129
Plasma	GSSG	0.008	0.041	0.843	−0.073	0.089
Plasma	GSH/GSSG Ratio	0.013	0.046	0.775	−0.079	0.106
Plasma	Total glutathione	0.016	0.049	0.74	−0.080	0.113
RBC Lysate	GSH	0.049	0.021	0.022	0.007	0.092
RBC Lysate	GSSG	0.064	0.031	0.044	0.002	0.126
RBC Lysate	GSH/GSSG Ratio	−0.015	0.108	0.891	−0.229	0.200
RBC Lysate	Total glutathione	0.050	0.022	0.023	0.007	0.093

## Data Availability

Restrictions apply to the availability of these data. Data was obtained by the study sponsor and may be available only with the written permission of study sponsor.

## Appendix A

**Table A1:** Recipe details of study foods.

**Table A2:** Nutritional composition of the study foods.

**Table A3:** Complete serum chemistry results.

**Table A4:** Complete CBC results.

**Figure A1:** Bodyweights of animals in all food groups during the test phase of the study.

**Figure A2:** Food intake of animals in all food groups during the test phase of the study.

**Figure A3:** Mean a-LA concentration in ppm in the different foods analyzed by High-Pressure Liquid Chromatography.

**Table A1:** Recipe details of study foods. Control, Food 1, Food 2 and Food 3 have 0 ppm, 75 ppm, 150 ppm and 300 ppm of a-LA respectively.

Ingredient	Food			
	Control	Food 1	Food 2	Food 3
Barley	11.9	11.9	11.9	11.9
Calcium carbonate	1.0	1.0	1.0	1.0
Chicken Meal	11.9	11.9	11.9	11.9
Choline chloride		0.20	0.20	0.20
Corn gluten meal	6.1	6.0	5.7	5.4
Corn	11.9	11.9	11.9	11.9
Fish oil	1.8	1.8	1.8	1.8
Lactic acid		1.2	1.2	1.2
Alpha Lipoic acid, 5%		0.20	0.40	0.78
Mineral Premix,	0.03	0.04	0.04	0.04
Flavor,	6.00	6.00	6.00	6.00
Pork Fat	5.0	5.0	5.0	5.0
Potassium chloride	0.41	0.41	0.41	0.41
Brewers Rice	11.9	11.9	11.9	11.9
Brown Rice	5.2	5.2	5.2	5.2
Iodized Sodium chloride	0.36	0.36	0.36	0.36
Sorghum	5.0	5.0	5.0	5.0
Soybean Oil	3.3	3.3	3.3	3.3
Soybean	5.0	5.0	5.0	5.0
Vitamin Premix	0.06	0.06	0.06	0.06
Wheat	11.9	11.9	11.9	11.9

Values shown as percentage of total recipe.

**Table A2:** Nutritional composition of the study foods on an As is basis. Control, Food 1, Food 2 and Food 3 have 0 ppm, 75 ppm, 150 ppm and 300 ppm of a-LA, respectively.

Nutrient	Food			
	Control	Food 1	Food 2	Food 3
alpha-Lipoic acid, ppm	0.0	69.1	158.2	286.1
Ash, %	5.0	4.8	5.1	5.2
Diet Metabolic Energy, kcal/kg	3881	3814	3908	3836
Chloride, %	0.58	0.60	0.61	0.63
Fat Crude, %	15	15	15	15
Polyunsaturated fatty acids, %	8.7%	8.6%	8.5%	9.1%
Fiber Crude, %	2.7	2.7	2.6	2.7
Fiber Total Dietary, %	8.3	8.8	9.4	9.3
Magnesium, %	0.10	0.09	0.10	0.10
Moisture, %	9.4	9.3	9.4	9.0
Phosphorus, %	0.66	0.64	0.66	0.67
Potassium, %	0.59	0.55	0.58	0.60
Protein Crude, %	20	21	20	20
Sodium, %	0.3	0.28	0.31	0.3
Selenium, ppm	0.51	0.45	0.45	0.56
Vitamin C as AscorbicAcid, ppm	18	<17.2	<17.2	<17.2
Vitamin E—Total Tocopherols, IU/kg	108	111	98	110

**Table A3:** Complete serum chemistry results.

Analyte	Time Point	Control	Food 1	Food 2	Food 3
Alb/Glob Normal range 1.1–2.0	P1	1.6 ± 0.24	1.5 ± 0.22	1.5 ± 0.24	1.5 ± 0.24
	Month 0	1.7 ± 0.27	1.6 ± 0.23	1.6 ± 0.26	1.6 ± 0.48
	Month 6	1.6 ± 0.43	1.6 ± 0.28	1.6 ± 0.27	1.6 ± 0.25
Albumin (g/dL) Normal range 2.9–4.0	P1	3.6 ± 0.33	3.7 ± 0.29	3.6 ± 0.27	3.6 ± 0.28
	Month 0	3.8 ± 0.29	3.9 ± 0.23	3.8 ± 0.29	3.9 ± 0.25
	Month 6	3.7 ± 0.9	3.9 ± 0.28	3.8 ± 0.35	3.9 ± 0.23
ALP (U/L) Normal range 30–439	P1	102 ± 102	78 ± 102	72 ± 117	72 ± 68
	Month 0	149 ± 206	116 ± 167	106 ± 200	96 ± 130
	Month 6	194 ± 285	150 ± 238	124 ± 233	138 ± 210
ALT (U/L) Normal range 22–268	P1	52 ± 54	33 ± 11.1	38 ± 11.8	43 ± 32
	Month 0	37 ± 10.4	36 ± 21	45 ± 39	36 ± 23
	Month 6	55 ± 68	37 ± 13.1	40 ± 23	38 ± 22
Total Bilirubin (mg/dL) Normal range 0.0–35.1	P1	0.06 ± 0.05	0.08 ± 0.06	0.06 ± 0.05	0.07 ± 0.05
	Month 0	0.09 ± 0.04	0.09 ± 0.04	0.09 ± 0.05	0.07 ± 0.05
	Month 6	0.09 ± 0.03	0.1 ± 0.04	0.09 ± 0.05	0.09 ± 0.05
BUN (mg/dL) Normal range 6.40–20.50	P1	13.7 ± 2.9	13.8 ± 2.3	13.4 ± 3.6	14 ± 2.9
	Month 0	12.2 ± 3.2	12.1 ± 2.3	11.6 ± 2.8	12.2 ± 2.3
	Month 6	13.2 ± 3.5	13.1 ± 2.4	11.9 ± 2.1	13.8 ± 2.6
Bun/Creat. Normal range 10.30–33.60	P1	20 ± 5.9	21 ± 4.5	21 ± 5.6	22 ± 4.5
	Month 0	19.2 ± 5.4	19 ± 4	18.8 ± 3.9	20 ± 4.6
	Month 6	20 ± 5.2	20 ± 3.9	19.9 ± 5.1	22 ± 5.3
Calcium (mg/dL) Normal range 8.7–12.0	P1	10.3 ± 0.4	10.2 ± 0.49	10.2 ± 0.37	10.2 ± 0.55
	Month 0	10.3 ± 0.44	10.6 ± 0.49	10.3 ± 0.4	10.4 ± 0.61
	Month 6	10.4 ± 0.52	10.4 ± 0.49	10.3 ± 0.4	10.2 ± 0.61
Chloride (mmol/L) Normal range 105–115	P1	110 ± 2.5	110 ± 2.1	110 ± 1.9	109 ± 2
	Month 0	112 ± 2.5	113 ± 2.4	113 ± 2.2	112 ± 2
	Month 6	108 ± 1.9	109 ± 1.7	106 ± 10.6	108 ± 3.3
Cholesterol (mg/dL) Normal range 133–401	P1	203 ± 44	202 ± 38	202 ± 32	186 ± 36
	Month 0	215 ± 64	212 ± 50	215 ± 46	195 ± 37
	Month 6	213 ± 61	208 ± 48	219 ± 52	191 ± 37
Creatinine (mg/dL) Normal range 0.41–0.82	P1	0.71 ± 0.16	0.69 ± 0.1	0.68 ± 0.19	0.65 ± 0.11
	Month 0	0.66 ± 0.14	0.66 ± 0.1	0.64 ± 0.21	0.62 ± 0.12
	Month 6	0.66 ± 0.13	0.66 ± 0.18	0.63 ± 0.19	0.64 ± 0.13
Globulin (g/dL) Normal range 1.9–2.9	P1	2.4 ± 0.27	2.5 ± 0.28	2.4 ± 0.29	2.5 ± 0.44
	Month 0	2.3 ± 0.25	2.5 ± 0.29	2.4 ± 0.29	2.6 ± 0.8
	Month 6	2.6 ± 0.94	2.4 ± 0.29	2.4 ± 0.32	2.5 ± 0.37
Glucose (mg/dL Normal range) 58–96	P1	84 ± 11.3	83 ± 8.1	85 ± 9	82 ± 9.5
	Month 0	84 ± 8.1	76 ± 10	79 ± 9.9	80 ± 7.5
	Month 6	87 ± 10	85 ± 10	85 ± 11	86 ± 10.1
Magnesium (mg/dL) Normal range 1.7–2.3	P1	2 ± 0.13	2 ± 0.13	2.1 ± 0.19	2.1 ± 0.13
	Month 0	2.1 ± 0.14	2 ± 0.1	2.1 ± 0.15	2.1 ± 0.17
	Month 6	1.9 ± 0.13	1.9 ± 0.2	2 ± 0.13	2 ± 0.15
NA:K Normal range 28–37	P1	30 ± 1.6	31 ± 1	31 ± 2.6	31 ± 1.8
	Month 0	31 ± 2.4	31 ± 1.6	31 ± 1.7	31 ± 1.4
	Month 6	31 ± 1.7	32 ± 2.6	31 ± 2	32 ± 3
Phosphorus (mg/dL) Normal range 2.9–6.1	P1	4.4 ± 0.73	4.2 ± 0.68	4.5 ± 0.57	4.2 ± 0.72
	Month 0	4.1 ± 0.55	4.1 ± 0.65	4.3 ± 0.55	4 ± 0.39
	Month 6	4.3 ± 0.47	4 ± 0.57	4.3 ± 0.71	4.1 ± 0.56
Potassium (mmol/L) Normal range 4–5.3	P1	4.9 ± 0.25	4.8 ± 0.19	4.9 ± 0.42	4.8 ± 0.24
	Month 0	4.8 ± 0.38	4.8 ± 0.23	4.8 ± 0.26	4.7 ± 0.23
	Month 6	4.9 ± 0.24	4.7 ± 0.35	4.8 ± 0.53	4.7 ± 0.39
Total Protein (g/dL) Normal range 5.1–6.5	P1	6 ± 0.41	6.1 ± 0.38	6.1 ± 0.36	6.2 ± 0.49
	Month 0	6.1 ± 0.32	6.3 ± 0.29	6.1 ± 0.41	6.3 ± 0.33
	Month 6	6.3 ± 0.44	6.3 ± 0.3	6.2 ± 0.43	6.4 ± 0.41
Sodium (mmol/L) Normal range 144–150	P1	149 ± 1.3	149 ± 1.6	150 ± 1.8	148 ± 1.7
	Month 0	149 ± 2.1	150 ± 2	149 ± 1.4	148 ± 1.8
	Month 6	149 ± 1.8	150 ± 15	147 ± 13.6	149 ± 1.9
Triglycerides (mg/dL) Normal range 34–429	P1	107 ± 87	124 ± 107	78 ± 41	80 ± 59
	Month 0	94 ± 78	89 ± 53	88 ± 50	64 ± 17.1
	Month 6	98 ± 75	91 ± 61	85 ± 63	67 ± 24

Data are represented as mean ± standard deviation. P1 = beginning of pre-feeding phase, Month 0 = beginning of the feeding phase and, Month 6 = end of the feeding phase. Control, Food 1, Food 2 and Food 3 have 0 ppm, 75 ppm, 150 ppm and 300 ppm of a-LA, respectively.

**Table A4:** Complete CBC results.

Analyte	Phase	Control	Food 1	Food 2	Food 3
Basophils (%)	P1	0.19 ± 0.12	0.09 ± 0.1	0.15 ± 0.09	0.14 ± 0.10
	Month 0	0.12 ± 0.06	0.09 ± 0.08	0.11 ± 0.10	0.11 ± 0.07
	Month 6	0.09 ± 0.09	0.1 ± 0.08	0.11 ± 0.15	0.09 ± 0.07
Absolute Basophils (k/µL) Normal range 0.00–0.10	P1	0.01 ± 0.01	0.01 ± 0.01	0.01 ± 0.01	0.01 ± 0.01
	Month 0	0.01 ± 0.01	0.01 ± 0.01	0.01 ± 0.01	0.01 ± 0.01
	Month 6	0.01 ± 0.01	0.01 ± 0.01	0.01 ± 0.01	0.01 ± 0.01
Eosinophils (%)	P1	5.3 ± 2.5	6.2 ± 4.3	5.5 ± 2.4	6.7 ± 5.1
	Month 0	4.7 ± 2.4	5.3 ± 3	4.1 ± 1.6	7.2 ± 5.9
	Month 6	4.4 ± 2.2	4.6 ± 2.6	3.8 ± 1.5	6.7 ± 4.8
Absolute Eosinophils (k/µL) Normal range 0.00–1.40	P1	0.44 ± 0.31	0.5 ± 0.38	0.44 ± 0.24	0.63 ± 0.66
	Month 0	0.38 ± 0.19	0.43 ± 0.28	0.34 ± 0.18	0.68 ± 0.78
	Month 6	0.33 ± 0.16	0.36 ± 0.21	0.31 ± 0.18	0.59 ± 0.55
HCT (%) Normal range 33.0–58.7	P1	52 ± 3.7	50 ± 3.7	51 ± 4.3	51 ± 3.7
	Month 0	49 ± 4.5	50 ± 5.2	49 ± 3.3	50 ± 4.1
	Month 6	49 ± 3.3	49 ± 4.6	49 ± 5.4	48 ± 4.7
HGB (g/dL) Normal range 10.5–20.1	P1	17.2 ± 1.3	16.6 ± 1.2	17 ± 1.5	17 ± 1.3
	Month 0	16.8 ± 1.6	16.9 ± 1.7	16.7 ± 1.3	16.9 ± 1.5
	Month 6	16.3 ± 1.2	16.2 ± 1.6	16.4 ± 1.8	16 ± 1.7
Lymphocytes (%)	P1	27 ± 6	26 ± 7	26 ± 6	26 ± 6
	Month 0	24 ± 6	25 ± 7	24 ± 6	24 ± 6
	Month 6	23 ± 6	22 ± 7	23 ± 7	22 ± 5
Absolute Lymphocytes (k/µL) Normal range 0.30–3.90	P1	2.1 ± 0.4	2.1 ± 0.9	1.9 ± 0.5	2.2 ± 0.7
	Month 0	2 ± 0.6	2.1 ± 0.9	1.9 ± 0.5	2.1 ± 0.8
	Month 6	1.8 ± 0.7	1.8 ± 0.9	1.7 ± 0.5	1.9 ± 0.7
MCH (pg) Normal range 21.0–27.0	P1	23 ± 0.87	23 ± 0.71	23 ± 0.84	23 ± 0.87
	Month 0	23 ± 1.00	23 ± 1.00	23 ± 0.92	23 ± 0.77
	Month 6	23 ± 0.95	23 ± 0.83	23 ± 0.95	23 ± 0.79
MCHC (g/dL)Normal range 30.1–41.9	P1	33 ± 0.54	33 ± 0.86	33 ± 0.65	33 ± 0.56
	Month 0	34 ± 0.67	34 ± 1.20	34 ± 0.70	34 ± 0.65
	Month 6	33 ± 0.64	33 ± 0.69	33 ± 0.7	33 ± 0.68
MCV (fL) Normal range 63.0–78.3	P1	69 ± 2.9	70 ± 3.1	69 ± 3.1	69 ± 3.2
	Month 0	68 ± 3.0	68 ± 2.9	69 ± 3.1	69 ± 2.8
	Month 6	68 ± 2.8	69 ± 2.7	69 ± 3.4	69 ± 2.9
Monocytes (%)	P1	6.1 ± 1.3	5.8 ± 1.8	6.7 ± 2.7	5.6 ± 1.6
	Month 0	6 ± 1.1	6.4 ± 1.6	6.8 ± 2.2	6.3 ± 1.6
	Month 6	5.4 ± 1.4	5.5 ± 1.8	6 ± 2.3	5.8 ± 1.5
Absolute Monocytes (k/µL) Normal range 0.00–1.40	P1	0.47 ± 0.12	0.48 ± 0.23	0.49 ± 0.18	0.5 ± 0.23
	Month 0	0.5 ± 0.12	0.53 ± 0.22	0.53 ± 0.23	0.54 ± 0.16
	Month 6	0.42 ± 0.15	0.45 ± 0.17	0.46 ± 0.23	0.49 ± 0.16
Neutrophils (%)	P1	61 ± 5	62 ± 9	61 ± 7	62 ± 8
	Month 0	65 ± 7	63 ± 8	65 ± 7	63 ± 9
	Month 6	67 ± 7	68± 8	68 ± 9	66 ± 8
Absolute Neutrophils (k/µL) Normal range 2.5–15.7	P1	4.8 ± 1.4	5.1 ± 1.5	4.7 ± 1.6	5.4 ± 2.0
	Month 0	5.4 ± 1.1	5.3 ± 1.9	5.3 ± 1.7	5.4 ± 1.4
	Month 6	5.2 ± 1.4	5.7 ± 2.1	5.4 ± 2.0	5.6 ± 1.7
Platelets (k/µL) Normal range 140–540	P1	274 ± 73	270 ± 83	305 ± 95	315 ± 78
	Month 0	300 ± 86	265 ± 103	319 ± 104	312 ± 83
	Month 6	294 ± 103	277 ± 98	343 ± 113	313 ± 110
RBC (M/µL) Normal range 4.48–8.53	P1	7.6 ± 0.67	7.2 ± 0.51	7.5 ± 0.66	7.4 ± 0.63
	Month 0	7.3 ± 0.68	7.3 ± 0.81	7.2 ± 0.63	7.2 ± 0.75
	Month 6	7.2 ± 0.58	7.1 ± 0.76	7.2 ± 0.81	7 ± 0.85
RDW (fL) Normal range 12.9–21.0	P1	36 ± 1.2	37 ± 7.4	36 ± 1.4	36 ± 1.5
	Month 0	36 ± 1.3	37 ± 6.6	35 ± 1.4	35 ± 1.5
	Month 6	36 ± 1.4	36 ± 1.4	36 ± 1.7	35 ± 1.3
Reticulocytes (%) Normal range 0.31–1.58	P1	0.69 ± 0.41	0.64 ± 0.27	0.58 ± 0.23	0.98 ± 0.48
	Month 0	0.70 ± 0.21	0.83 ± 0.42	0.58 ± 0.2	0.80 ± 0.36
	Month 6	0.79 ± 0.31	0.77 ± 0.35	0.71 ± 0.34	0.77 ± 0.38
Absolute Reticulocytes (M/ul)	P1	0.05 ± 0.03	0.05 ± 0.02	0.04 ± 0.02	0.07 ± 0.04
	Month 0	0.05 ± 0.02	0.06 ± 0.03	0.04 ± 0.02	0.06 ± 0.03
	Month 6	0.06 ± 0.02	0.06 ± 0.03	0.05 ± 0.03	0.05 ± 0.03
WBC (ku/µL Normal range) 4.0–18.2	P1	7.8 ± 1.8	8.2 ± 2.1	7.6 ± 2.2	8.7 ± 2.8
	Month 0	8.3 ± 1.4	8.3 ± 2.7	8.1 ± 2.2	8.7 ± 2.3
	Month 6	7.8 ± 1.9	8.3 ± 2.6	7.9 ± 2.6	8.5 ± 2.5

Data are represented as mean ± standard deviation. P1 = beginning of pre-feeding phase, Month 0 = beginning of the feeding phase and, Month 6 = end of the feeding phase. Control, Food 1, Food 2 and Food 3 have 0 ppm, 75 ppm, 150 ppm and 300 ppm of a-LA, respectively.
